# Metabolomic profiles in breast cancer:a pilot case-control study in the breast cancer family registry

**DOI:** 10.1186/s12885-018-4437-z

**Published:** 2018-05-05

**Authors:** Marcelle M. Dougan, Yuqing Li, Lisa W. Chu, Robert W. Haile, Alice S. Whittemore, Summer S. Han, Steven C. Moore, Joshua N. Sampson, Irene L. Andrulis, Esther M. John, Ann W. Hsing

**Affiliations:** 1 Stanford Cancer Institute, Stanford University School of Medicine, Stanford, California, USA; 2 San Jose State University, San Jose, CA USA; 3 Cancer Prevention Institute of California, Fremont, California, USA; 4 Cedars-Sinai Medical Center, Los Angeles, CA USA; 5 National Cancer Institute, Bethesda, MD USA; 6 Lunenfeld-Tanenbaum Research Institute, Sinai Health System, University of Toronto, Toronto, ON Canada; 7 Stanford Prevention Research Center, Stanford University School of Medicine, Stanford, California, USA

## Abstract

**Background:**

Metabolomics is emerging as an important tool for detecting differences between diseased and non-diseased individuals. However, prospective studies are limited.

**Methods:**

We examined the detectability, reliability, and distribution of metabolites measured in pre-diagnostic plasma samples in a pilot study of women enrolled in the Northern California site of the Breast Cancer Family Registry. The study included 45 cases diagnosed with breast cancer at least one year after the blood draw, and 45 controls. Controls were matched on age (within 5 years), family status, *BRCA* status, and menopausal status. Duplicate samples were included for reliability assessment. We used a liquid chromatography/gas chromatography mass spectrometer platform to measure metabolites. We calculated intraclass correlations (ICCs) among duplicate samples, and coefficients of variation (CVs) across metabolites.

**Results:**

Of the 661 named metabolites detected, 338 (51%) were found in all samples, and 490 (74%) in more than 80% of samples. The median ICC between duplicates was 0.96 (25th – 75th percentile: 0.82–0.99). We observed a greater than 20% case-control difference in 24 metabolites (*p* < 0.05), although these associations were not significant after adjusting for multiple comparisons.

**Conclusions:**

These data show that assays are reproducible for many metabolites, there is a minimal laboratory variation for the same sample, and a large between-person variation. Despite small sample size, differences between cases and controls in some metabolites suggest that a well-powered large-scale study is likely to detect biological meaningful differences to provide a better understanding of breast cancer etiology.

**Electronic supplementary material:**

The online version of this article (10.1186/s12885-018-4437-z) contains supplementary material, which is available to authorized users.

## Background

Metabolomics is the systematic survey of the small molecules (< 1 k Dalton in size) that are the products of metabolism in biological systems [1, 2]. A metabolic phenotype represents the collection of metabolites within the body which reflects influences from both genetic and lifestyle/environmental factors. Because metabolites include the intermediate- and end-products of the cellular processes, metabolomics provides a functional readout of the physiological state of health and disease. Changes in energy metabolism within cells are one of the hallmarks of carcinogenesis. Under aerobic conditions, normal cells metabolize energy by first converting glucose into pyruvate and then to carbon dioxide, and under anaerobic conditions, cells metabolize by glycolysis. However, the converse is true for cancer cells, where under aerobic conditions, energy metabolism occurs largely by glycolysis, i.e., “aerobic glycolysis” [3]. Thus, a characterization of metabolic processes may provide new insights into carcinogenesis. In recent years, metabolomics has emerged as an important tool for the identification of biomarkers in a growing number of applications, including early disease detection, monitoring of disease progression, and investigation of metabolic pathways. The application of metabolomics has yielded novel signatures predicting the occurrence and progression of complex diseases, including cancers of the breast [4], prostate, colon, and kidney [5–8].

Most metabolomics studies of breast cancer to date have been conducted in tumor tissues or cell lines and with the goals of distinguishing cancer from normal tissue and cancers with metastasis from those without, as well as identifying therapeutic targets [9–11]. Data from these studies have suggested that metabolomic profiles may differ by pathological and molecular subtype of breast cancer. In large scale epidemiologic studies, blood and urine are more readily available than tissue. Because blood and urine serve as transporters of nutrients and wastes to and from cells for excretion, and maintain homeostasis of essential molecules and fluid levels, they are sensitive indicators of health and perturbations from diseases. Several studies have measured urinary metabolic profiles and found promising candidate markers for early detection and monitoring of breast cancer progression [12–14]. However, studies using pre-diagnostic blood are limited. To our knowledge, there has been only one previously published study on metabolomics and breast cancer risk using pre-diagnostic blood [4], warranting additional studies to replicate the findings in other populations. We conducted a pilot study to generate preliminary data to assess whether circulating metabolomic profiles could be detected in pre-diagnostic plasma samples of women enrolled in the Breast Cancer Family Registry (BCFR) cohort, and to evaluate the reproducibility of metabolomic assays.

## Methods

### Study population

Pre-diagnostic plasma samples were obtained from the BCFR, an international prospective cohort of breast cancer families established in 1995 [15] [16]. For this pilot study, samples were selected from the Northern California site (NC-BCFR), which enrolled women with newly diagnosed breast cancer (probands) identified through the population-based cancer registry of the San Francisco Bay area and family members [17]. At baseline, participants completed a risk factor questionnaire and provided a blood sample. During follow-up, newly diagnosed breast cancer cases were identified among family members who were unaffected at baseline. This pilot study included 45 women who were diagnosed with breast cancer at least one year after the blood draw (cases) and 45 women who did not develop breast cancer (controls). Of the 45 cases, 72% of the cases were confirmed via cancer registry linkage or pathology reports; the remainder were self-reported. Controls were matched to cases on family status (a sister was selected if available; if more than one sister was available, we selected the sister closest in age), age at blood draw (±5 years), menopausal status at diagnosis, and number of affected first degree relatives (1, 2, or ≥3). The age range for cases was 26–80 years (average 52.4 years), and that for controls was 36–73 years (average 53 years).

### Laboratory assays

Plasma samples obtained from cases and matched controls were aliquoted into 200 μl ethylenediaminetetraacetic acid (EDTA) plasma vials. Case-control sets were assayed in the same batch and adjacent to each other in sequence. Samples were identified by specimen ID only, and laboratory technicians were masked to the case-control status of samples. The samples were assayed on the Discovery HD4 platform, a mass spectrometry-based metabolomics profiling platform, at Metabolon (Durham, NC, USA). This method combines automated sample extraction processing, an ultrahigh performance liquid chromatography/electrospray ionization tandem mass spectrometry (UHPLC/MS) with additional gas chromatography mass spectrometry (GC/MS) platform. Peaks were quantified using area-under-the-curve and metabolite levels were generated. The metabolite data were normalized to a median of 1.00 to correct for variation resulting from instrument tuning differences. Metabolites not detected in individual samples were imputed with the minimum value for that metabolite. The data were then log-transformed to reduce non-normality. Duplicates from 10 controls were included to assess assay reproducibility. Data from the duplicates were used to assess intra-class correlation (ICC) and were averaged for the case-control analysis.

### Statistical analysis

To assess the reliability of our assay results, we calculated coefficients of variation (CVs) and ICCs across duplicate samples. Coefficient of variation is a measure of dispersion, that describes the amount of variability relative to the mean. For samples measured using the same method, a low (~ 10%) variability within subjects and high variability across subjects is desirable. Intra-class correlations describe the degree to which duplicate samples agree: a value between 0.75 and 1 indicates excellent agreement.

We used the variance component from a one-way analysis of variance (ANOVA) model to estimate the ICC for replicate samples, and estimated confidence intervals for the ICCs using the Smith method [18] in R (ICC-Package) [19]. We calculated CVs across named metabolites, and used principal component analysis [20] to identify the important components (groups of metabolites) in each sample (including the duplicates). We used paired t-tests to examine differences in the normalized metabolite levels between cases and controls. We used non-linear modeling to examine whether normalized metabolite levels were associated with age at blood draw. We evaluated quadratic and cubic models and used the Akaike Information Criterion (AIC) to evaluate the best model fit. We also evaluated using ANOVA with robust variance to examine differences in metabolite levels by the number of affected first-degree relatives (1, 2, or ≥3), and among cases with available information, by estrogen receptor (ER) status (positive/negative) and progesterone receptor (PR) status (positive/negative). Due to the limited sample size of this pilot study, further analysis of subgroups by age was not statistically meaningful.

## Results

Of the 45 cases selected, 31 had two or more affected first-degree female relatives, while 14 cases had one affected first-degree female relative. Six cases were *BRCA1* mutation carriers, while four were *BRCA2* mutation carriers. Twenty-one cases were premenopausal at blood collection, and the remainder were postmenopausal. The average age at breast cancer diagnosis was 58.8 years. The average age at blood draw was 52.4 years for cases, compared to 53.1 years for controls. Approximately 51% of cases were ER positive, and 22% were ER negative. About 20% of cases had localized tumors, and 5% had regional involvement limited to the nodes (Table 1).

**Table 1:** Characteristics of Study Subjects

	Cases (*n* = 45)	Controls (*n* = 45)
Age at blood draw, years	52.4 (26–80)	53 (36–73)
Age at diagnosis, years	58.8 (30–84)	N/A
Post-menopausal at blood draw, %	58	60
Number of affected first-degree relatives, %		
1	29	31
2	29	27
3	42	42
*BRCA1* Mutation, %		
Positive	7	9
Negative	20	13
Unknown	73	78
*BRCA2* Mutation, %		
Positive	2	11
Negative	11	9
vUnknown	87	80
Estrogen Receptor Status, %		
Positive	51	–
Negative	22	-
Unknown	27	
Progesterone Receptor Status, %		
Positive	53	–
Negative	20	-
Unknown	27	
Human Epidermal Growth Factor Receptor 2 Status, %		
Positive	9	-
Negative	60	–
Unknown	31	
Summary Stage, %		
Localized	20	–
Regional, nodes only	11	–
Unknown	69	

*Values are means (range) or percentages

We detected a total of 661 known named metabolites in our samples. Of these, 338 (51%) were detected in all the samples, and 490 (74%) were detected in greater than 80% of the 90 study samples. These metabolites include amino acids and lipids, and some related to microbiome influences and xenobiotics metabolism (Additional file 1: Table S1). The average CV across all named metabolites was 0.16 (25th – 75th percentile: 0.06–0.20) (Table 2). The median ICC between duplicates was 0.96 (25th – 75th percentile: 0.82–0.99). The average variance was 60.7% among individuals, and 6.0% for duplicate samples within individuals.

**Table 2:** Distribution of observed coefficient of variation for named and most common^1^ metabolites among 45 cases and 45 controls

	Named Metabolites	Most common^1^ metabolites
N	661	490
Average	0.16	0.132
Median	0.11	0.099
Standard deviation	0.18	0.11
Confidence interval?	0.01	0.01
Lower 95% CI	0.14	0.12
Upper 95% CI	0.17	0.14
1st quartile	0.06	0.06
2nd quartile	0.11	0.10
3rd quartile	0.20	0.16
4th quartile	2.00	0.89

^1^Present in > 80% of samples

**Fig. 1:**
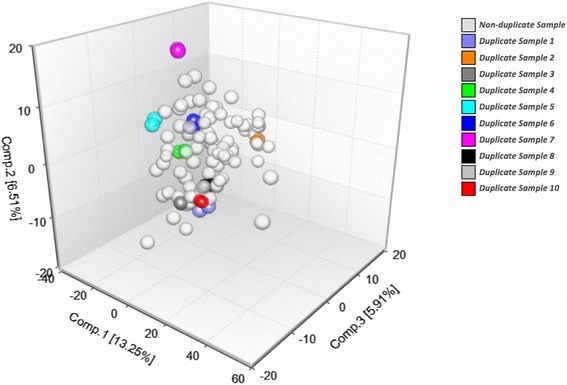
Top three components identified by Principal Component Analysis

Principal component analysis identified the top 3 components of all samples. The scores of the components identified were very similar for duplicate samples. (Fig. 1).

**Fig. 2:**
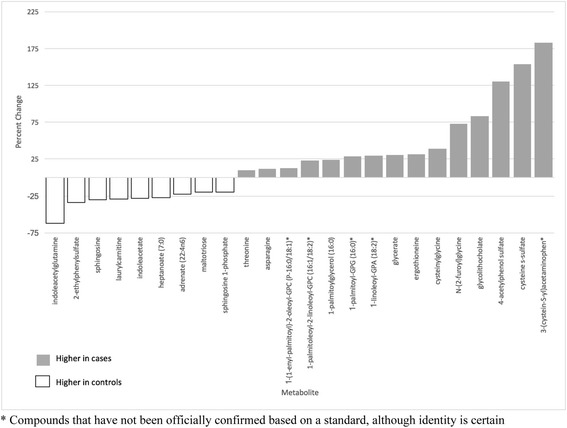
Differences in metabolites between cases and controls (*p* < 0.05)

We observed a greater than 20% case-control difference in 24 metabolites that were statistically significant (*p* < 0.05). Metabolites including 3-(cystein-S-yl)acetaminophen (xenobiotics pathway), 4-acetylphenol sulfate (xenobiotics pathway), and cysteine s-sulfate (amino acid pathway) were significantly higher in cases, whereas indoleacetylglutamine, (amino acid pathway), 2-ethylphenylsulfate (xenobiotics pathway), and sphingosine (lipid pathway) were significantly higher in controls (Fig. 2).

Among the metabolites that showed a greater than 20% case-control difference, we also examined differences among hypothesized predictors of breast cancer risk, including differences by age at blood draw, the number of affected first-degree relatives (Table 3), ER status, and PR status (Table 4). Statistically significant (*p* < 0.05) but modest associations were observed between some metabolites and age at blood draw; for example, age explained 13% of the variation in 1-(1-enyl-palmitoyl)-2-oleoyl-GPC (P-16:0/18:1) (adjusted r^2^ = 0.13; *p* < 0.01).

**Table 3:** Differences by key breast cancer risk factors among metabolites that showed a greater than 20% case-control difference

Metabolite	Metabolism Pathway	Age at blood draw^a^ (Adjusted R^2^)		Number of affected first-degree relatives mean levels (standard deviation)			
		*n* = 90	*P*-value^b^	1 (*n* = 27)	2 (*n* = 25)	3 (*n* = 38)	*P*-value^b^
1-(1-enyl-palmitoyl)-2-oleoyl-GPC (P-16:0/18:1)^3^	Plasmalogen	0.13	**< 0.01**	1.02 (0.24)	0.98 (0.27)	1.16 (0.38)	**0.02**
1-linoleoyl-GPA (18:2) ^c^	Lysolipid	0.01	0.29	0.97 (0.51)	1.22 (0.64)	1.39 (1.11)	0.06
1-palmitoleoyl-2-linoleoyl-GPC (16:1/18:2) ^3^	Phospholipid	0.01	0.23	1.15 (0.56)	1.04 (0.42)	1.01 (0.45)	0.75
1-palmitoyl-GPG (16:0) ^c^	Lysolipid	< 0.01	0.92	0.78 (0.53)	0.93 (0.98)	1.05 (0.91)	0.23
1-palmitoylglycerol (16:0)	Monoacylglycerol	0.07	**0.02**	1.07 (0.39)	0.93 (0.31)	1.22 (0.59)	**0.001**
2-ethylphenylsulfate	Benzoate	< 0.01	0.38	0.68 (0.51)	0.97 (0.92)	0.64 (0.51)	**0.001**
3-(cystein-S-yl)acetaminophen^c^	Xanthine	< 0.01	0.96	0.50 (1.08)	0.22 (0.08)	0.47 (1.38)	0.12
4-acetylphenol sulfate	Drug	0.01	0.71	0.82 (0.64)	1.24 (2.78)	1.60 (3.83)	0.35
adrenate (22:4n6)	Polyunsaturated fatty acid (n3 and n6)	< 0.01	0.78	1.28 (0.61)	0.83 (0.62)	1.13 (0.66)	0.78
asparagine	Alanine and aspartate	0.04	0.06	1.01 (0.32)	1.14 (0.39)	1.08 (0.27)	0.15
cysteine s-sulfate	Glycogen	0.09	**0.01**	3.81 (6.30)	4.89 (6.51)	4.28 (7.47)	0.8
cysteinylglycine	Glutathione	0.01	0.24	1.05 (0.50)	1.10 (0.68)	1.15 (0.67)	0.2
ergothioneine	Food component/plant	< 0.01	0.36	1.24 (0.91)	1.04 (0.55)	1.35 (1.46)	0.21
glycerate	Glycolysis, Gluconeogenesis, and Pyruvate	< 0.01	0.57	1.09 (0.45)	1.35 (0.94)	1.23 (0.56)	**0.03**
glycolithocholate	Secondary bile acid	0.01	0.20	0.94 (1.08)	1.03 (1.78)	0.82 (0.98)	0.66
heptanoate (7:0)	Medium chain fatty acid	< 0.01	0.64	1.06 (1.15)	0.86 (1.07)	0.72 (0.54)	0.24
indoleacetate	Tryptophan	0.01	0.57	1.23 (0.75)	1.43 (0.98)	1.19 (0.89)	0.71
indoleacetylglutamine	Tryptophan	< 0.01	0.39	1.75 (6.23)	0.77 (0.89)	0.74 (0.56)	0.05
laurylcarnitine	Fatty acid (acyl carnitine)	0.03	0.12	1.62 (2.24)	0.92 (0.60)	1.15 (0.66)	0.08
maltotriose	Glycogen	0.04	0.10	1.17 (0.51)	1.05 (0.56)	0.93 (0.47)	0.92
N-(2-furoyl)glycine	Food component/plant	0.02	0.38	3.87 (7.12)	1.62 (3.61)	4.07 (6.35)	**0.03**
sphingosine	Sphingolipid	< 0.01	0.62	1.03 (0.60)	0.91 (0.53)	1.12 (1.29)	0.06
sphingosine 1-phosphate	Sphingolipid	0.03	0.12	1.08 (0.49)	0.98 (0.41)	0.98 (0.50)	0.32
threonine	Glycine, serine, and threonine	0.05	0.05	0.97 (0.22)	1.03 (0.32)	1.05 (0.22)	0.07

^a^The age models for all metabolites were fitted with a linear and a quadratic term, with the exception of maltitriose, which also included a cubic term for age, as the model fit was improved for this metabolite over the liner and quadratic model

^b^Not adjusted for multiple comparisons

^c^Indicates compounds that have not been officially confirmed based on a standard, although identity is certain

Significant data are in bold

**Table 4:** Differences by key breast cancer variables among metabolites that showed a greater than 20% case-control difference

Metabolite	Metabolism Pathway	ER Status mean levels (standard deviation)			PR Status mean levels (standard deviation)		
		ER + (*n* = 23)	ER - (*n* = 10)	*P*-value^1^	PR + (*n* = 24)	PR - (n = 9)	*P*-value^a^
1-(1-enyl-palmitoyl)-2-oleoyl-GPC (P-16:0/18:1) ^b^	Plasmalogen	1.16 (0.26)	1.26 (0.35)	0.14	1.15 (0.27)	1.29 (0.32)	0.75
1-linoleoyl-GPA (18:2) ^b^	Lysolipid	1.50 (1.03)	1.21 (0.62)	0.25	1.54 (0.99)	1.08 (0.63)	0.3
1-palmitoleoyl-2-linoleoyl-GPC (16:1/18:2) ^b^	Phospholipid	1.19 (0.60)	1.26 (0.40)	0.52	1.22 (0.60)	1.21 (0.41)	0.59
1-palmitoyl-GPG (16:0) ^2^	Lysolipid	1.08 (0.89)	0.76 (0.41)	0.11	1.06 (0.88)	0.80 (0.40)	0.1
1-palmitoylglycerol (16:0)	Monoacylglycerol	1.19 (0.45)	1.07 (0.35)	0.62	1.17 (0.47)	1.13 (0.29)	0.11
2-ethylphenylsulfate	Benzoate	0.63 (0.63)	0.59 (0.52)	0.53	0.65 (0.62)	0.55 (0.53)	0.51
3-(cystein-S-yl)acetaminophen^2^	Xanthine	0.92 (2.06)	0.36 (0.34)	0.07	0.89 (2.02)	0.38 (0.35)	0.1
4-acetylphenol sulfate	Drug	1.23 (1.96)	1.31 (1.00)	0.39	1.98 (4.78)	0.98 (0.40)	0.29
adrenate (22:4n6)	Polyunsaturated fatty acid (n3 and n6)	1.03 (0.65)	0.80 (0.48)	0.21	1.07 (0.66)	0.69 (0.32)	0.04
asparagine	Alanine and aspartate	1.11 (0.24)	1.19 (0.43)	0.1	1.12 (0.24)	1.19 (0.46)	**0.02**
cysteine s-sulfate	Glycogen	6.17 (8.69)	4.92 (7.29)	0.44	6.34 (8.80)	4.33 (6.56)	0.25
cysteinylglycine	Glutathione	1.23 (0.67)	1.28 (0.62)	0.56	1.27 (0.65)	1.19 (0.69)	0.9
ergothioneine	Food component/plant	1.55 (1.41)	1.27 (0.85)	0.45	1.57 (1.39)	1.18 (0.82)	0.36
glycerate	Glycolysis, Gluconeogenesis, and Pyruvate	1.23 (0.40)	1.26 (0.61)	0.2	1.23 (0.40)	1.24 (0.63)	0.25
glycolithocholate	Secondary bile acid	2.01 (4.89)	1.01 (0.42)	0.24	1.31 (1.92)	1.13 (1.03)	0.44
heptanoate (7:0)	Medium chain fatty acid	0.88 (1.22)	0.75 (0.51)	0.41	0.65 (0.68)	1.33 (1.64)	0.08
indoleacetate	Tryptophan	1.02 (0.53)	1.24 (0.92)	0.64	1.11 (0.74)	1.03 (0.41)	**0.03**
indoleacetylglutamine	Tryptophan	0.46 (0.06)	0.54 (0.31)	**0.01**	0.50 (0.21)	0.44 (0)	0.12
laurylcarnitine	Fatty acid (acyl carnitine)	1.15 (0.86)	0.82 (0.45)	0.04	0.20 (0.82)	0.69 (0.46)	0.05
maltotriose	Glycogen	0.94 (0.49)	0.97 (0.44)	0.8	0.88 (0.42)	1.13 (0.55)	0.44
N-(2-furoyl)glycine	Food component/plant	3.46 (5.33)	5.71 (6.53)	0.72	2.87 (4.18)	7.55 (7.90)	0.04
sphingosine	Sphingolipid	0.83 (0.52)	0.83 (0.68)	0.11	0.84 (0.55)	0.80 (0.63)	0.69
sphingosine 1-phosphate	Sphingolipid	0.94 (0.46)	0.94 (0.39)	0.37	0.92 (0.46)	0.99 (0.39)	0.42
threonine	Glycine, serine, and threonine	1.08 (0.22)	1.10 (0.30)	0.31	1.09 (0.26)	1.08 (0.19)	0.46

^a^Not adjusted for multiple comparisons

^b^Indicates compounds that have not been officially confirmed based on a standard, although identity is certain

Significant data are in bold

We examined whether metabolites differed by the number of affected first-degree relatives. Overall, there was no clear monotonically increasing or decreasing pattern by the number of affected relatives: mean levels were similar for women with one or three affected relatives, and lower or higher for those with two affected relatives. For example, for 2-ethylphenylsulfate, the mean levels were 0.68, 0.97, and 0.64, for women with one, two, or three affected relatives, respectively (*p* < 0.001).

In some metabolites, we found differences by ER status. For example, cases with ER+ breast cancer had higher mean laurylcarnitine level than those with ER- breast cancer (1.16 vs. 0.83, *p* = 0.04). Conversely, the mean indoleacetyleglutamine level was lower for ER+ breast cancer cases than ER- cases (0.46 vs. 0.54, *p* = 0.04).

Finally, we examined whether metabolite levels differed by PR status. The mean asparagine level for PR+ cases was 1.12, compared to 1.18 for PR- cases (*p* = 0.02). For adrenate (22:4n6), the mean level for PR+ cases was 1.07 compared to 0.69 for PR- cases, and for N-(2-furoyl)glycine, the mean level for PR+ cases was 2.87 compared to 7.54 for PR- cases. However, none of the associations remained significant after adjusting for multiple comparisons.

## Discussion

These data, despite small numbers, suggest that a large number of metabolites have detectable levels, with good reproducibility, as suggested by high ICCs and reasonable CVs. We also showed that for most metabolites, the within-person variance is small, while the between-person variance is much larger. We found that some metabolites have a greater than 20% case-control difference. Finally, we showed that some metabolites (including N-(2-furoyl)glycine in the xenobiotics pathway) differed by key breast cancer risk factors such as the number of affected family members, although these associations were not significant after adjusting for multiple comparisons, likely due to the small sample size. Taken together, these results suggest that a large-scale study (~ 1000) would be well-powered to detect meaningful biological and statistically significant differences between cases and controls to provide a better understanding of breast cancer etiology across a wide spectrum of risks, and among high-risk women in particular.

Metabolomics profiles are becoming increasingly utilized in epidemiological studies to predict the risk of chronic diseases, including breast cancer; however, data from prospective studies are limited. In the first prospective study of metabolomics and breast cancer risk, Kuhn et al. [4] found that phosphatidylcholines were associated with breast cancer risk. That study included 362 sporadic breast cancer cases, and measured only 120 metabolites. To date, there are no metabolomics data on women at increased risk of breast cancer due to their family history of breast cancer. Clearly additional data from prospective studies are needed to further examine the role of metabolomics in breast carcinogenesis.

The assay performance on our samples, measured by ICCs and CVs, is consistent with earlier studies that have examined the utility of metabolomics in epidemiological research among participants of the Shanghai Physical Activity Study [21]. In that study, the variability in a large subset of metabolites was assessed and the intraclass correlation was high (median 0.8). Similar assay performance was also observed in a nested case-control study of metabolomics and colorectal cancer risk [22] that included 254 cases and 254 matched controls from the Prostate, Lung, Colorectal and Ovarian Cancer study. In that study, which used a metabolomics platform similar to the one used in our pilot study, the median intraclass correlation was 0.86 (25th–75th percentile: 0.64–0.92).

Consistent with our observation that age at blood collection was associated with metabolite levels, Saito et al. [23] also reported that certain metabolites were associated with age at blood draw in a Japanese population. Because the populations in the two studies are quite different, a direct comparison is not possible. Similarly, Tang et al. [24] reported that metabolites in tumor tissues were associated with ER status, and also with *BRCA1*-associated tumors. However, studies utilizing human plasma are limited.

One notable limitation of our study is that we did not match on the duration of storage time between cases and controls. Post-hoc analysis revealed that among case-control pairs, 35 pairs (78%) had a difference of less than 3 years of storage duration, while 1 pair had a difference of more than 10 years. Further analyses showed that while there were no appreciable differences in the analyses by age, there were differences in 5 metabolites when evaluating levels by the number of affected relatives. Future studies should match on calendar year of blood draw (hence storage duration) within case-control matched sets.

Our study is among the first to examine the association between metabolomics and breast cancer risk using pre-diagnostic plasma samples. Despite the limited sample size, we were able to find a larger than 20% case-control difference in several metabolites, although we cannot rule out the possibility that the presence of asymptomatic preclinical breast cancer may have affected metabolite levels in cases. Such bias is possible but should be minimal as we excluded cases diagnosed with breast cancer within 12 months after the blood draw in order to limit the potential for preclinical disease to influence metabolite levels. Finally, our study is also among the first to examine reliability across more than 600 metabolites.

## Conclusions

In conclusion, findings from this study suggest that metabolomics can be used reliably in large-scale epidemiologic studies of breast cancer to detect meaningful differences in risk.

## Additional file

Additional file 1: **TableS1.** Number of metabolites measured in plasma of BCFR participants (DOCX 17 kb)
