# Supplementary material for: Metabolomic profiles in breast cancer:a pilot case-control study in the breast cancer family registry
Source: BMC Cancer. 2018 May 5;18:532. doi: 10.1186/s12885-018-4437-z (PMC5935968; doi:10.1186/s12885-018-4437-z)
Supplement: Supplementary file 1 — TableS1. Number of metabolites measured in plasma of BCFR participants (DOCX 17 kb) [file 12885_2018_4437_MOESM1_ESM.docx]

**Supplemental** **Table 1. Number of metabolites measured in plasma of BCFR participants**

| **Category** | **Pathway** | **Number of metabolites** |
| --- | --- | --- |
| Amino Acids | Glycine, Serine and Threonine Metabolism | 9 |
|  | Alanine and Aspartate Metabolism | 6 |
|  | Glutamate Metabolism | 8 |
|  | Histidine Metabolism | 11 |
|  | Lysine Metabolism | 10 |
|  | Phenylalanine and Tyrosine Metabolism | 31 |
|  | Tryptophan Metabolism | 15 |
|  | Leucine, Isoleucine and Valine Metabolism | 25 |
|  | Methionine, Cysteine, SAM and Taurine Metabolism | 19 |
|  | Urea cycle; Arginine and Proline Metabolism | 14 |
|  | Creatine Metabolism | 3 |
|  | Polyamine Metabolism | 4 |
|  | Guanidino and Acetamido Metabolism | 2 |
|  | Glutathione Metabolism | 5 |
| Peptides | Gamma-glutamyl Amino Acid | 14 |
|  | Dipeptide Derivative | 1 |
|  | Dipeptide | 3 |
| Carbohydrates | Glycolysis, Gluconeogenesis, and Pyruvate Metabolism | 6 |
|  | Pentose Metabolism | 4 |
|  | Glycogen Metabolism | 2 |
|  | Disaccharides and Oligosaccharides | 1 |
|  | Fructose, Mannose and Galactose Metabolism | 3 |
|  | Aminosugar Metabolism | 3 |
|  | Advanced Glycation End-product | 1 |
| Energy | TCA Cycle | 10 |
|  | Oxidative Phosphorylation | 1 |
| Lipids | Medium Chain Fatty Acid | 8 |
|  | Long Chain Fatty Acid | 14 |
|  | Polyunsaturated Fatty Acid (n3 and n6) | 13 |
|  | Fatty Acid, Branched | 2 |
|  | Fatty Acid, Dicarboxylate | 10 |
|  | Fatty Acid, Amino | 2 |
|  | Fatty Acid Synthesis | 2 |
|  | Fatty Acid Metabolism (also BCAA Metabolism) | 4 |
|  | Fatty Acid Metabolism(Acyl Glycine) | 1 |
|  | Fatty Acid Metabolism(Acyl Carnitine) | 15 |
|  | Carnitine Metabolism | 2 |
|  | Ketone Bodies | 1 |
|  | Fatty Acid, Monohydroxy | 13 |
|  | Fatty Acid, Dihydroxy | 2 |
|  | Eicosanoid | 1 |
|  | Endocannabinoid | 5 |
|  | Inositol Metabolism | 1 |
|  | Phospholipid Metabolism | 33 |
|  | Lysolipid | 25 |
|  | Plasmalogen | 11 |
|  | Lysoplasmalogen | 4 |
|  | Glycerolipid Metabolism | 2 |
|  | Monoacylglycerol | 5 |
|  | Diacylglycerol | 6 |
|  | Sphingolipid Metabolism | 27 |
|  | Mevalonate Metabolism | 1 |
|  | Sterol | 3 |
|  | Steroid | 28 |
|  | Primary Bile Acid Metabolism | 7 |
|  | Secondary Bile Acid Metabolism | 13 |
| Nucleotides | Purine Metabolism, (Hypo)Xanthine/Inosine containing | 6 |
|  | Purine Metabolism, Adenine containing | 6 |
|  | Purine Metabolism, Guanine containing | 4 |
|  | Pyrimidine Metabolism, Orotate containing | 3 |
|  | Pyrimidine Metabolism, Uracil containing | 8 |
|  | Pyrimidine Metabolism, Cytidine containing | 1 |
|  | Pyrimidine Metabolism, Thymine containing | 2 |
| Cofactors and | Nicotinate and Nicotinamide Metabolism | 5 |
| Vitamins | Pantothenate and CoA Metabolism | 1 |
|  | Ascorbate and Aldarate Metabolism | 2 |
|  | Tocopherol Metabolism | 8 |
|  | Biotin Metabolism | 1 |
|  | Folate Metabolism | 1 |
|  | Hemoglobin and Porphyrin Metabolism | 6 |
|  | Vitamin A Metabolism | 1 |
|  | Vitamin B6 Metabolism | 2 |
| Xenobiotics | Benzoate Metabolism | 15 |
|  | Xanthine Metabolism | 15 |
|  | Tobacco Metabolite | 3 |
|  | Food Component/Plant | 31 |
|  | Drug | 51 |
|  | Chemical | 17 |
|  | **Total** | **661** |
